# Deep learning in breast cancer risk prediction: a review of recent applications in full-field digital mammography

**DOI:** 10.3389/fonc.2025.1656842

**Published:** 2025-09-03

**Authors:** João Mendes, Bernardo Oliveira, Carolina Araújo, Joana Galrão, Ana M. Mota, Nuno C. Garcia, Nuno Matela

**Affiliations:** ^1^ Instituto de Biofísica e Engenharia Biomédica, Faculdade de Ciências, Universidade de Lisboa, Lisboa, Portugal; ^2^ LASIGE, Faculdade de Ciências, Universidade de Lisboa, Lisboa, Portugal; ^3^ Departamento de Física, Faculdade de Ciências, Universidade de Lisboa, Lisboa, Portugal

**Keywords:** artificial intelligence, breast cancer, risk prediction, digital mammography, deep learning

## Abstract

Breast Cancer (BC) remains one of the most commonly diagnosed cancers worldwide. Even though standard screening procedures have made positive impacts on disease burden, their accuracy remains limited. Personalized screening, based on individual risk, offers the potential to improve disease outcomes. While traditional risk models based on well-established factors, such as age and family history, are widely used, their discriminatory power is still insufficient. Artificial Intelligence (AI), already playing a role in breast cancer diagnosis, has the potential to make an impact on the field of risk prediction. AI models that utilize imaging biomarkers could help create more personalized risk profiles, enabling clinicians to adapt screening either in terms of imaging modality used or periodicity. Moreover, it also enables women to make changes to their lifestyle in order to diminish their risk of BC development. Therefore, this review fills a gap in the literature by exploring recent advancements in AI risk prediction using imaging biomarkers from Full-Field Digital Mammography. Moreover, this work also addresses challenges that must be overcome before clinical implementation.

## 1 Introduction

### 1.1 Breast cancer facts, statistics, and diagnosis

In 2022, over 2.3 million cases of female Breast Cancer (BC) were diagnosed worldwide. In total, over 650 thousand women died from BC, making it the fourth deadliest cancer Bray et al. (1).Moreover, studies predict that by 2040, BC incidence will increase by 40% and mortality by 50% Arnold et al. (2). These projections highlight that BC is not only a current public health concern but will remain a leading cause of premature death in the future. The burden caused by this disease can be countered by promoting early diagnosis, potentially reducing BC death rates in the long term Wang (3). Early detection of BC can be promoted through generalized screening programs Ginsburg et al. (4), usually based in Full-field digital mammography (FFDM) Wang (3)that have shown to decrease mortality Nelson et al. (5). Nonetheless, these programs present some pitfalls, with data showing that, in the United States of America screened population, 1 in 8 cancers are missed Schaffter et al. (6). In addition, FFDM screening also presents some problems related to false positive results, negatively impacting woman’s mental well-being, and potentially diminishing trust on healthcare services Løberg et al. (7), besides contributing to unnecessary expenses with complementary examinations.

### 1.2 Risk factors

Several factors contribute to the increased risk of breast cancer in women. Some of them can be avoided, being related to lifestyle factors, and others are intrinsic to each woman, being non-modifiable. One of these is age, with studies finding that over 80% of the BC patients are above 50 years of age Łukasiewicz et al. (8). Besides aging, family history of BC also makes women more prone to the development of this disease Brewer et al. (9). Breast density is also a significant risk factor for breast cancer, with women who have denser breast tissue facing a higher risk of developing the disease Łukasiewicz et al. (8). This fact is particularly important given that FFDM has a decreased sensitivity for women with higher breast density Niell et al. (10), meaning that screening mammography performs worse for women at higher risk. Increased risk of BC is also observed in women with specific genetic mutations, such as BRCA1 and BRCA2 mutations Łukasiewicz et al. (8).

Regarding risk factors related to lifestyle changes, high body mass index seems to be associated with a higher risk of BC, at the same time that physical activity appears to have a protective role for BC incidence. Likewise, high alcohol intake and smoking habits might contribute to increase BC risk Łukasiewicz et al. (8). Besides that, prolonged exogenous exposure to estrogens via hormonal replacement therapy is also an important risk factor Sun et al. (11).

### 1.3 Risk prediction models

Considering the vast range of factors that contribute to the increase in BC risk, several models have been developed and used to predict the risk of being diagnosed with this disease. Stratifying women into risk groups is crucial, as it enables physicians to tailor screening protocols and follow-up strategies accordingly, while also empowering women to adopt lifestyle changes aimed at reducing their breast cancer risk. The Gail model Gail et al. (12) incorporates several factors, including current age, age at menarche, and age at first live birth. Even though it considers the family history of BC to predict risk, no information regarding BRCA1/2 mutations is considered. In opposition, the BRCAPRO model focuses on the presence of these specific mutations Engel and Fischer (13). Finally, the Tyrer-Cuzick model aims to predict the risk of BC using BRAC1/2 screening results alongside personal risk factors information (age, age at first menarche, body mass index, etc), and family history of both BC and ovarian cancer Himes et al. (14). Each of these models has limitations regarding its application. The Gail model does not accurately estimate risk for women with lobular carcinoma *in situ*, and it has been shown to underestimate BC risk for African American women. The BRCAPRO model may underperform when analyzing individuals from mixed-race families. The Tyrer-Cuzick model, on the other hand, typically overestimates risk for women with atypia Cintolo-Gonzalez et al. (15).

Nonetheless, despite ongoing efforts to refine risk prediction models, improvements in their discriminatory power and overall accuracy remain limited Louro et al. (16).

### 1.4 AI in breast cancer

Despite their shortcomings, the presented risk prediction models based on epidemiological risk factors are widely used in clinical practice. Given these limitations, recent expert discussions have advocated the integration of imaging biomarkers into risk prediction models as a means to enhance predictive performance Mendes et al. (17). Artificial Intelligence (AI) presents itself as a promising tool for the extraction and application of those imaging biomarkers. In fact, AI has been proven to effectively provide significant improvements to the field of BC imaging Mendes et al. (18). Concretely, AI systems developed with the goal of aiding in the diagnosis of BC have shown to increase diagnostic accuracy without increasing healthcare professionals’ workload Rodríguez-Ruiz et al. (19); Pacilè et al. (20).

The translation of knowledge from the aid in diagnosis with AI to the field of risk prediction can help to overcome some of the major challenges with current screening practice. Accurate breast cancer risk prediction enables personalized screening strategies, both in terms of imaging modality and screening intervals, potentially reducing missed diagnoses, improving patient prognosis, and ultimately lowering healthcare costs. Additionally, effective risk prediction models can promote greater equity in the access to quality healthcare. Since generalized screening programs are not universally available or uniformly implemented across countries Mendes et al. (17), risk-based approaches can help healthcare systems in resource-limited regions prioritize women at higher risk. This allows for better allocation of limited resources, ensuring that those most in need receive timely and appropriate screening.

Given the shortcomings of traditional risk models and the impact of AI on the field, we propose a focused review of the last five years of research on deep learning-based breast cancer risk prediction using mammography. This review aims to synthesize recent advancements in AI-based breast cancer risk prediction, evaluate their contribution to the field, and explore both their limitation and application in clinical practice. The paper is organized as follows: Section 2 describes the methodology used to identify relevant studies; Section 3 summarizes the selected works and their key characteristics; Section 4 discusses the main findings, addresses the current limitations and outlines; finally, Section 5 summarizes the key insights of this work.

## 2 Materials and methods

With the goal of identifying relevant studies for this research, a structured literature search was conducted on two databases (Scopus and Pubmed), with the goal of answering the following research questions:

- What are the recent advances in Deep Learning methodologies for BC risk prediction?
- What are the differences in Deep Learning performance from short-risk to long-risk prediction?
- What are the limitations on current Deep Learning methods for BC risk prediction?

With the goal of identifying novel approaches on BC risk prediction with Deep Learning, the following search was conducted: breast AND cancer AND risk AND prediction AND (“CNN” OR “deep learning”).

**Figure 1:**
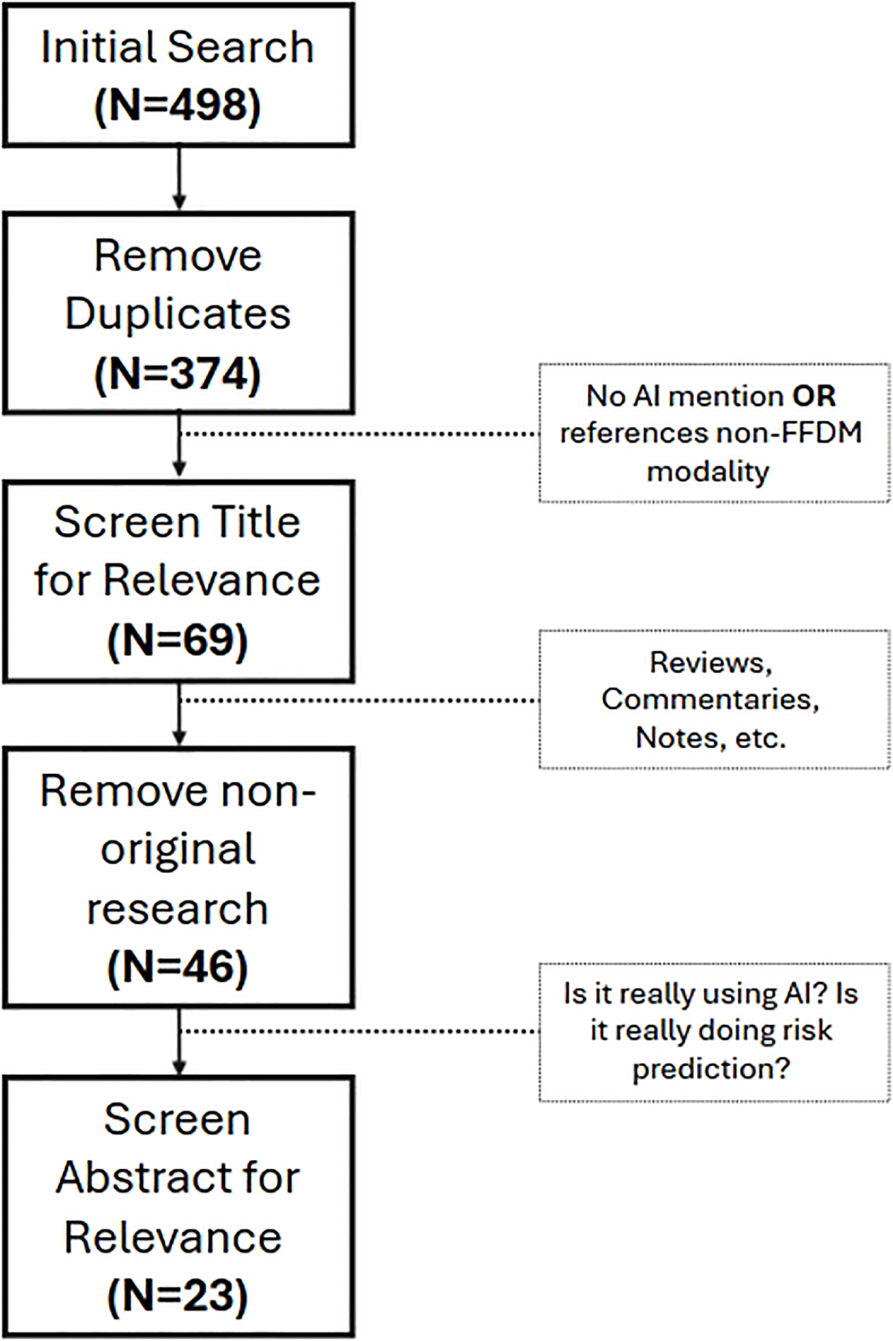
Screening process for paper inclusion on this review. First, duplicates were removed. Then, through title assessment, papers non-related to the research questions were removed. After that, non-original research papers were removed. Finally, the abstract of the 46 papers that remained were screened for relevance, resulting in 23 papers included in this review.

**Figure 2:**
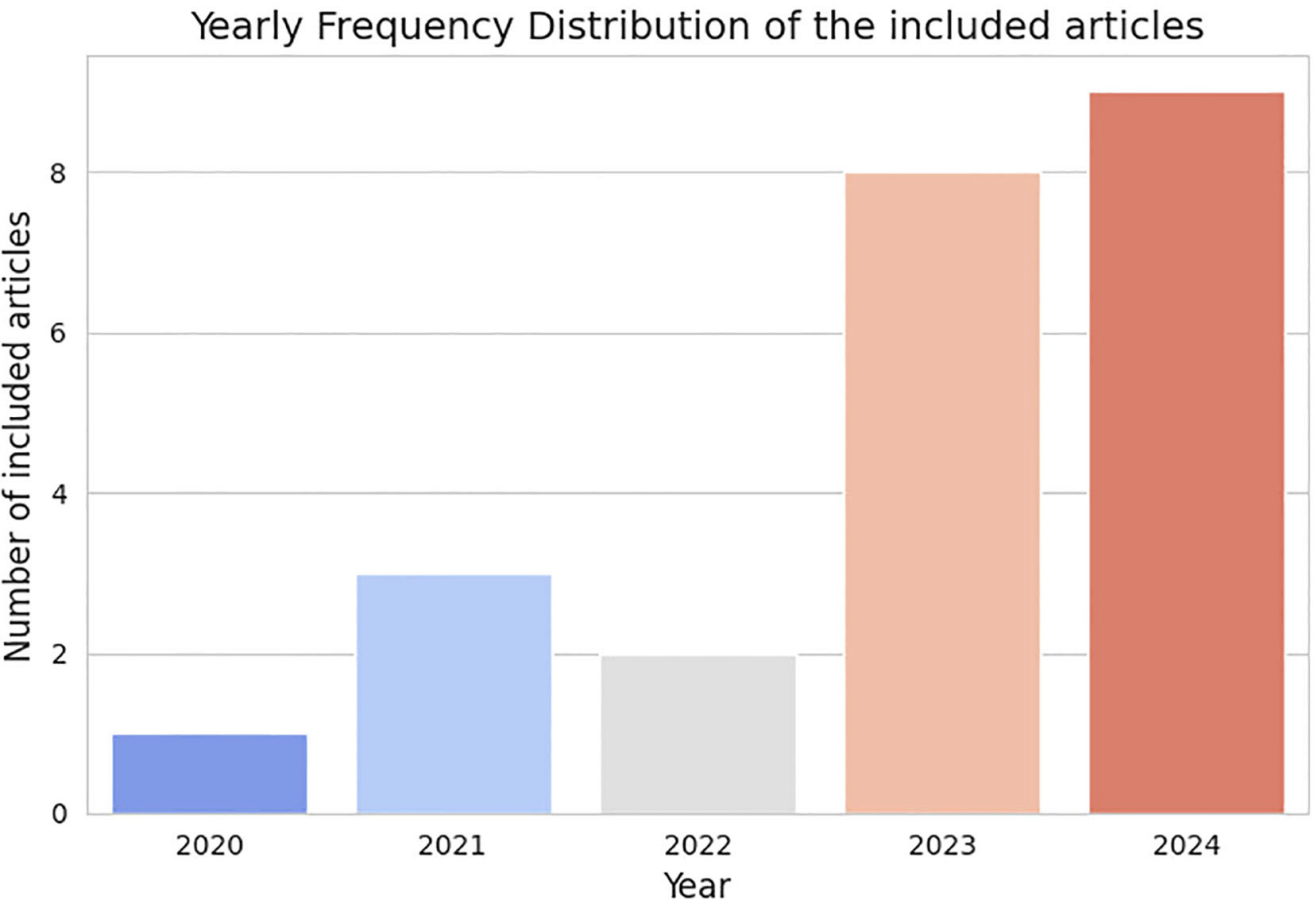
Distribution of papers by the year they were published.

Moreover, since the designed research focused on approaches from the last five years, the time-frame was limited from 2020 to 2024 (cutoff in September), with the fact that the papers were indexed on these databases serving as quality control. In total, 496 articles result from our search. After removing duplicates from both databases, 374 unique articles remained. In order to refine our search, all papers were screened based on the title. If it was clear that they did not match the purpose of this review or used imaging techniques that are not widely used for screening (e.g., MRI), the papers were removed. After this step, 69 articles remained as possibly relevant to be included on this review. Since our focus is only on original work, we removed every review paper, editorials and comments from the article pool, which reduced the database to 46 articles. Finally, all the abstracts were read and, if it was clear that they did not match the purpose of this review, they were not considered. After this thorough evaluation, 23 articles were selected and included in the final analysis. **Figure 1** represents this selection process, while **Figure 2**, shows the distribution of papers by the year they were published.

This approach ensured that the review was based on a well-defined dataset, accurately reflecting the intended scope of AI-based breast cancer risk prediction.

## 3 Results

### 3.1 5-year/long-term risk prediction

Several studies have investigated breast cancer risk prediction over specific time horizons. This section focuses on a 5 year time-frame.

**Table 1** summarizes the reviewed papers on this subsection, describing the dataset used, model’s backbone architecture, key findings and limitations of each research.

**Table 1:** Summary of 5-year/long-term breast cancer risk assessment studies.

Authors	Year	Data	Cohort type	Architecture	Main results	Key findings	Limitations
Yala et al. (21)	2021	Train: 205,440 risk-negative/5,379 risk-positive; Test: 13,112 risk-negative/244 risk-positive	Screening	MIRAI (ResNet- 18)	AUC= 0.79^*^	Outperforms traditional risk models and DL methods	Dataset lacks ethnic variability
Zhu et al., (22)	2021	Train: 2,760 risk-negative/1,046 risk-positive; Test: 907 risk-negative/431 risk-positive	Case- Control	DenseNet- 12	C-statistic =0.66	DL outperforms clinical factor models	Potential misclassificati of interval cancers
Kim et al., (23)	2023	Train: 506 risk-negative/195 risk-positive; Test: 199 risk-negative/43 risk-positive	Screening	Commercial software	AUC = 0.75	Reasonable risk prediction on dense- only data	Low sensitivity
Romanov et al. (24)	2024	Train: 2,364 risk-negative/788 risk-positive) Test: 320 risk-positive	Case- Control	ResNet- 18 + MIL + Attention	AUC = 0.62”	MIL reduces computation with fair performance	Does not exceed existing methods
Omoleye et al. (25)	2023	Test: 5,061 risk-negative/1,205 risk-positive	Case- Control	MIRAI	AUC = 0.65^*^	Better for short-term prediction	Self- reported race/ethnicity is limited
Donnelly et al., (26)	2024	Train: EMBED training cohort (Jeong et al. (27)); Test: 16,083 risk-negative/257 risk-positive	Screening and Diagnostic	MIRAI adaptation	AUC = 0.66	Bilateral dissimilarity predicts BC	Performance varies across racial subgroups
Klanecek et al. (28)	2024	Years to cancer: 0 - 1 (589), 1 - 3 (436), 3 - 5 (171), 5 - 7 (14)	Screening	MIRAI	AUC = 0.55 - 0.58 (3 – 5 yrs)	Short- term predictions focus on future tumor site	Small long-term sample

^*^Test set is external.

Yala et al. (21) developed a model called MIRAI (which has ResNet-18 as backbone), which, despite having a 5-year time horizon, can make predictions for each individual year within that period (1- to 5-year risk). The model extracts information from four mammography views, aggregates this information, and then applies an additive hazard layer. This layer combines mammography data with risk factor information (such as age and weight) to predict a patient’s risk for each of the next five years. For training, the authors used a dataset of 210,819 examinations, of which 5,379 were followed by a cancer diagnosis within five years. The test set included 25,855 examinations, with 588 identified as positive. These were screening examinations acquired at the Massachusetts General Hospital, using the Hologic system. While white women comprised nearly 80% of the training, African American and Asian or Pacific Islanders were each represented with almost 5% of the cases. The model achieved a 5-year Area Under the Curve (AUC) up to 0.79 on external test sets (with different characteristics from that used for training). Park et al. (29) aimed for a similar approach but with an higher case:control ratio. The obtained results were similar to those reported for MIRAI; however, direct comparisons between models are only valid when evaluated on the same benchmark dataset under similar conditions.

Zhu et al. (22) conducted a study to test whether deep learning models based on negative mammograms could more accurately predict the risk of cancer detected at screening. They compared this model (that uses DenseNet-12) to a logistic regression model using clinical risk factors and BI-RADS breast density, developed by the authors as a baseline comparator. Women aged 40 to 74 years with negative mammograms acquired between 6 months and 5 years before diagnosis were included. The mammograms used in this study were sourced from a non-profit academic center and from a medical center within a public research university. Both locations are in the United States of America. It was concluded that the deep learning model outperformed the risk factor and density model for cancers detected at screening (C-statistic: 0.66 vs. 0.62). The authors also conducted experiments on the prediction of interval cancers, but their model was outperformed by traditional risk factors. In this study, the C-statistic serves a similar role to the AUC, indicating the model’s ability to discriminate between cases and controls.

Kim et al. (23) conducted a study involving 1023 images, 287 of which were from women at risk of breast cancer, with the aim of developing a deep learning model based solely on mammograms to predict breast cancer risk in Asian women. The images were acquired at two different settings, using Hologic and GE HealthCare machinery. The model was trained at two levels: the exam level, which included two images per breast, and the single image level. In both cases, the sensitivity (ability to identify the positive class) was very low (*<*50%). The precision at the image level was 30.4%, and at the exam level it was 52.6%. These metrics present very low values, with the high accuracy being attributed to the significant data imbalance.

In Romanov et al. (24), Romanov et al. analyzed a total of 3,152 mammograms, including 788 from women with a history of breast cancer (2,364 controls remained cancer free). Among these, 487 cases were cancers detected during screening, while 301 were interval cancers (detected between mammograms). To develop the model, 868 cases were included, 400 of which were screening-detected cancers, with the remaining being previous cancers. For each case, three matched controls were used. This study utilized data from women invited to screening in the Greater Manchester area, with the diversity of the dataset limited by the characteristics of the population served by the healthcare facilities of this region. Each image was divided into 244x244 patches, a methodology designed to eliminate differences between CC and MLO views, treating them equally. ResNet-18 was utilized to extract features from each patch, creating a final aggregated vector for the prediction phase. A conditional model was also developed, incorporating age, BMI (Body Mass Index), menopausal status, and HRT (Hormone Replacement Therapy). This model achieved an AUC of 0.620, with a comparable performance to common risk prediction methods. Notably, the model performed better in predicting interval cancers than cancers detected during screening.

In Omoleye et al. (25), Omoleye et al. aims to validate the MIRAI model using a cohort of ethnically diverse women. In addition, the authors seek to investigate whether the risk predictions made by the model are influenced by age or breast density. The images used in this study represent the population served by the Cancer Risk Clinic or the Breast Center at the University of Chicago. The dataset is nearly evenly divided between White women (41.8%) and African American women (44.5%), and also includes Asian or Pacific Islander, Hispanic, and Alaska native women, though in smaller proportions. Upon testing the Mirai model, the authors found that for 1-year risk prediction, the model achieved an AUC of 0.71, while the value for 5-year prediction was 0.65. However, after removing cases where cancer was diagnosed within 6 months of the last examination, these values dropped to 0.64 and 0.63, respectively. No statistical differences were found between age or race groups. However, the authors did observe that the model performed slightly better for non-dense breasts. Overall, when testing the Mirai model on this ethnically diverse dataset, the performance was below that of other state-of-the-art works. This study has several limitations, such as the almost exclusive consideration of White and African American women.

Donnelly et al. (26) proposed a simple risk prediction model based on the differences between the right and left mammograms of a patient. The mammograms used in the study were input into a ResNet-18 model for feature extraction for each view. The authors describe the use of the EMory BrEast imaging Dataset, that contains data from African American, American Indian or Alaskan Native, Asian, White, Native Hawaiian or Pacific Islander women. The mammograms present on this dataset were acquired with Hologic, GE Healthcare, and Fujifilm machinery. The authors then computed localized bilateral dissimilarity at multiple locations. The maximum dissimilarity value across these locations was used to produce a dissimilarity score, which was averaged across views to generate a single bilateral dissimilarity score, considered as the risk score. This architecture allows the outputs to be directly overlaid on the input mammogram. Using this approach, the authors achieved an AUC of 0.79 for 1-year risk prediction and 0.66 for 5-year risk prediction. The fact that this method relies on dissimilarity and needs to use the four mammographic views might make its use computationally heavy. However, since screening routines already consist of the acquisition of these views, the model is capable of leveraging all the available information in clinical practice to make a decision.

Klanecek et al. (28) conducted this study to explore the functioning of the MIRAI model and its dependence on the evolving characteristics of the breast tissue where cancer developed. The study analyzed 1210 FFDM screening images acquired 0 to 7 years before cancer detection, with Hologic machinery. The distribution of cases was as follows: 589 cases from 0 – 1 year, 436 cases from 1 – 3 years, 171 cases from 3 – 5 years, and 14 cases from 5 – 7 years. To investigate the influence of breast characteristics on classification, seven interpretability models were applied to the predictions of the MIRAI model. An innovative technique, Information Bottlenecks for Attribution, was adapted for 4-dimensional inputs (2 breasts × 2 views). Attribution maps were calculated for each mammogram, summed separately for the left and right breasts, and the side with the highest value was considered to contribute more to the classification. The results revealed that the earlier the mammogram was acquired, the less the model became dependent on the characteristics of the breast associated with cancer. For the 0 – 1 year group, this dependence was strong but diminished over time, with a significant drop in the 5 – 7 year group, suggesting a shift toward randomness. However, the small sample size of the study, particularly the 14 cases in the 5 – 7 year group, represents a notable limitation.

### 3.2 4-year risk prediction

**Table 2** summarizes the reviewed papers on this subsection.

**Table 2:** Summary of 4-year breast cancer risk assessment studies.

Authors	Year	Data	Cohort type	Architecture	Main results	Key findings	Limitations
Wagner et al., (30)	2024	Train: 80% of 846 participants: 423 risk-negative/423 risk-positive Test: 10% of 846 participants: 423 risk-negative/423 risk-positive	Screening	Ensemble + Autoencoder + LSTM	AUC = 0.74	Longitudinal mammograms may improve prediction	Retrospective data increases computational burden
Lee et al. (31)	2023	Train: 10,988 risk-negative (includes normal and benign outcomes)/3,125 Test: 898 risk-negative/302 risk-positive	Screening and Diagnostic	ResNet- 34	AUC = 0.76	Prior images improve risk prediction	Limited performance if density changes across years
Dadsetan et al., (32)	2022	5-fold Cross-validation: 100 risk-negative/100 risk-positive	Case- Control	VGG-16 + GRU	AUC = 0.67	Spatiotemporal breast tissue changes are predictive	Small dataset

If we consider a 4-year time horizon for predicting BC, we can refer to Wagner et al. (30), who used transfer learning from a detection model Frazer et al. (33) to enhance risk prediction. Their approach leveraged longitudinal data from the same women. The images were sourced from the Flemish screening program and acquired with one of the following Siemens equipment: Mammomat Novation DR, Mammomat Inspiration, Mammomat Revelation. To reduce feature dimensionality, they employed an autoencoder, and for predicting 4-year BC risk, they used a Long Short-Term Memory (LSTM) to effectively process the sequential nature of their data. Including not only the most recent (cancer-free) examination but also prior exams led to an AUC of 0.74. In contrast, in a separate study Wagner et al. (34), the authors applied a similar method but relied only on the most recent exam, achieving a slightly higher AUC of 0.77. Both models outperformed the Tyrer-Cuzick model, demonstrating the benefit of Deep Learning (DL) approaches in BC risk prediction.

The aim of Lee et al. (31) was to develop a convolutional neural network (CNN) that uses pre-cancer mammograms in order to assess the risk of developing BC through attentional models. The authors trained and evaluated the model on a dataset of more than 9000 patients. The validation set contained 800 scans from 400 patients: 198 cancer scans, 210 benign scans and 392 normal scans. The test set contained 1200 scans from 600 patients: 302 cancer scans, 290 benign and 608 normal. To improve risk prediction, the authors combined information from previous and current mammograms. The images were all driven from institutions across the United States of America and Driven from Hologic and Siemens devices. ResNet-34 was used as a feature extractor. The authors reached several conclusions focusing on time-dependent AUC, with the use of previous mammograms improving the obtained results. The same occurs with the use of the transformer decoder, used to integrate relevant information from both images before the risk prediction. The highest AUC value (0.76) was obtained for a 2-year prediction after excluding cases less than 180 days old. However, overall, the model showed a time-dependent drop in AUC due to the exclusion of these cases.

Dadsetan et al. (32) aimed to develop a deep learning model that could learn the spatial and temporal variations of breast tissue. In order to do that, a study was carried out based on the analysis of negative sequential mammograms of 200 women. The mammograms were acquired using Hologic/Lorad Selenia mammography units. The authors consider the use of 4 prior mammograms to predict the “current” status. A CNN that detects changes in breast tissue was developed. With the model using all the 4 priors, an AUC of 0.67 was obtained, the best result compared to other ablation studies carried out (using less than 4 priors, or using each prior as single inputs). Finally, the authors demonstrated that the simultaneous use of CC and MLO views has a great advantage for the model when compared to models that use only one of the views.

### 3.3 3-year risk prediction

**Table 3** summarizes the reviewed papers on this subsection.

**Table 3:** Summary of 3-year breast cancer risk assessment studies.

Authors	Year	Data	Cohort type	Architecture	Main results	Key findings	Limitations
Dadsetan et al. (35)	2021	Train: 60% of 306 participants: 153 risk-negative/153 risk-positive Test: 20% of 306 participants: 153 risk-negative/153 risk-positive	Screening	Inception- ResNetV2	AUC = 0.66	Prior mammograms improve risk prediction	Small dataset
Romanov et al. (36)	2023	Train: 3798 risk-negative/1266 risk-positive (includes biopsy proven screen-detected cancers) Test: 960 risk-negative /320 risk-positive	Screening	ResNet- 18 + MIL + Attention	AUC = 0.75^*^	MAI model outperforms traditional risk models	Global feature extraction is limited
Santeramo et al. (37)	2024	Test: 1,693 risk-negative/1,693 risk-positive)	Screening	Four open access algorithms	AUC = 0.67 (MIRAI)	Detection improvement aid risk prediction	Black-box s models; screening program limitations
Ellis et al. (38)	2024	Train: 86,127 risk-negative/3,158 risk-positive Test: 38,298 risk-negative/1,053 risk-positive	Screening	ShuffleNet	AUC = 0.70	DL captures non- density patterns	Only singleview images used
Wang et al. (39)	2024	Development: 50 risk-negative/risk-positive	Screening	MIRAI	AUC = 0.74	Calcification features predictive of early BC	Small dataset

^*^Test set is external.

This section addresses studies aimed at predicting breast cancer risk within a three-year timeframe. In 2021, Dadsetan et al. (35) tested and compared different methods to predict the future risk of breast cancer based on prior mammograms. To achieve this, three Deep Learning systems for risk prediction in 1, 2 and 3 years were developed. In a second task, a 3-year prediction model was first trained using transfer learning from ImageNet. Then, a 2-year model was developed using transfer learning from the 3-year model. Finally, a 1-year models was trained using transfer learning fom the 2-year model. The best result was obtained when using the 2-year model to make 1-year risk prediction (AUC = 0.66). Finally, Multi-task learning was conducted, where the three individual models (one for each year) shared weights across all layers except for the last one, which is task-specific. The conclusions were that incorporation of prior mammograms generally promotes better performance and that the closer the prior image is to a diagnosis, the better the model ‘s performance. This study highlights the advantage of incorporating longitudinal information in risk prediction.

Romanov et al. (36) developed an attention-based Multiple Instance Learning (MIL) model capable of making short-term risk predictions from mammograms performed 5 to 55 weeks prior the cancer detection. The mammograms were driven from another study Astley et al. (40) and were acquired using GE Senographe Essential mammography unit. The results of this study showed a great capacity to predict cancer detection (AUC = 0.75), however, due to the fact that MIL considers only small patches of the breast at a time, the general characteristics of the breast tissue tend to be lost.

Santeramo et al. (37), building upon their previous UK validation of the MIRAI model, took a different approach by verifying whether algorithms that perform well in cancer detection yield the same results in cancer risk prediction. Three open access models specialized on detection were used on this study, while MIRAI was used as the model specialized in risk prediction. In this study, paired mammograms were used from the same women: one taken at the time of diagnosis for detection models and the other three years earlier, cancer-free, for prediction models. The study included 1693 controls, equal to the number of cases. The included patients attended the National Health Service Breast Cancer Screening Program and had their mammograms acquired with a Hologic unit. The used data is part of the OPTIMAM dataset Halling-Brown et al. (41). Four different algorithms were applied: one for risk prediction and the others initially designed for detection, all using CNNs. The performance of the four models was evaluated for both detection and prediction, and it was concluded that, for the controls, there was a moderate to good correlation between the performance of the models on the task of detection and risk prediction. For the cases, the correlation was also moderate to good, however, greater variation was observed. The model designed for risk prediction was the one that showed the best performance both in risk (AUC = 0.67) and detection. Therefore, this analysis suggests that the better the algorithm’s performance in cancer detection, the better its performance in risk prediction.

The aim of Ellis et al.’s (38) work was to develop a deep learning-based AI tool capable of predicting future breast cancer risk, up to 3 years in advance, using a current negative screening mammogram. The OPTIMAM dataset Halling-Brown et al. (41) was used on this study and only cases acquired on Hologic machinery were considered. The dataset was mostly composed of images from white women but also contained, in lower proportions, images from Asian and Black women. The AI model, based on a pre-trained ShuffleNet, achieved an AUC of 0.70, significantly outperformed a model using age as the only variable (AUC = 0.56). However, this model used only one manufacturer and a single view per patient.

Wang et al. (39) aimed to assess whether prior mammograms could be used to predict the current BI-RADS (Breast Imaging-Reporting and Data System) assessment. The results show that the extracted calcification features demonstrated the ability to classify the current BI-RADS analysis with an AUC of 0.74.

### 3.4 Broad risk assessment

**Table 4** summarizes the reviewed papers on this subsection.

**Table 4:** Summary of broad breast cancer risk assessment studies.

Authors	Year	Data	Cohort type	Architecture	Main results	Key findings	Limitations
Melek et al. (42)	2023	5-fold cross-validation: 130 risk-negative/61 risk-positive	Screening	Selfdeveloped CNN (Siamese Network)	AUC = 0.81	Multi-timepoint mammograms improve risk prediction	Single-institution study; small dataset
Michel et al. (43)	2023	Test: 23,346 risk-negative/121 risk-positive	Screening	Selfdeveloped CNN	AUC = 0.654 (CNN + clinical)	Large and ethnically diverse dataset	Limited number of invasive cancers
Li et al (44)	2023	5-fold cross-validation: 50 risk-negative/49 risk-positive	Screening	VGG-19 + LSTM	AUC = 0.65 (CNN + radiomics)	Radiomics + CNN are useful for risk prediction	Small cohort; varied intervals; manual ROI alignment
Lauritzen et al. (45)	2023	Train: 37,922 risk-negative/1,423 risk-positive (includes screen-detected, long-term and interval cancers) Test: 117,030 risk-negative/2,620 risk-positive	Screening	SE-ResNet-18	AUC = 0.73	Diagnostic AI may aid BC risk prediction	Density distributions differ between datasets
Dembrower et al. (46)	2020	Test: 2,005 risk-negative/278 risk-positive	Screening	Inception- ResNetV2	AUC = 0.65	DL can learn beyond density only features	No external validation

Some studies take a more general approach, predicting breast cancer risk without focusing on a specific time horizon. Melek et al. (42), used a Siamese network, consisting of two identical networks with shared weights. Each network received mammograms from two different time points (T1 and T2, with T2 *>* T1) to assess if spatiotemporal data improved risk prediction. Their dataset included 191 participants, 61 of whom later developed cancer. The Siamese model outperformed using T1 or T2 alone (AUC = 0.81 vs 0.75 and 0.77), though F1-scores were modest (0.61 vs. 0.54 and 0.59). Mohamed et al. (47) took a similar approach but instead of feeding mammograms from different time points into the Siamese network, they used prior mammograms from both the left and right breasts. Their bilateral analysis proved more effective than unilateral risk prediction, achieving hiher AUC (0.75 vs 0.70).

Michel et al. (43) developed a CNN model based on U-net to predict risk using prior mammmograms acquired at least on year before cancer was diagnosed. The images belong to women that were screened at the Columbia University Irving Medical Center in New York City. The dataset contains images mostly from White and Hispanic women, but it also contains data from Black and Asian women. They used a logistic regression model to combine the CNN risk score with clinical factors. This hybrid model, despite achieving a higher AUC (0.654 vs. 0.624) than a model only using risk factors, did not achieve a statistically significant higher performance. Despite using a large ethnically variable dataset, only a small number of cancer cases were included on the study which might have pushed down the results.

Li et al. (44) also evaluated the use of multiple time points for BC risk prediction, comparing it to a single-time-point model. The screening images belong to women who attended the MD Anderson Cancer Center and the University of Chicago Medical Center and were all acquired using Hologic units. They extracted deep (VGG - 19) and radiomic features from a region behind the nipple. Longitudinal data were processed with an LSTM, while single time data were classified using a Support Vector Machine (SVM). Their findings showed that longitudinal models generally outperformed single-time-point approaches. The best performance (AUC = 0.65) was achieved by combining CNN and radiomics from both breasts of the same woman, though similar results were found using only radiomics extracted from the breast that later developed cancer. Nonetheless, the participants had different follow-up periods, and the ROIs were not spatially aligned.

Lauritzen et al. (45), using images acquired with Mammomat Inspiration system, combined a commercial detection model for short-term risk (examination score) with a custom texture model for long-term risk (texture risk). These scores, along with age and mammographic density, were used to generate a final risk score. To better understand risk, they differentiated interval and long-term cancer cases from those who remained cancer free. The combined scores outperformed individual models (AUC = 0.73 vs 0.70 for examination and 0.66 for texture), indicating that integrating lesion detection models enhances risk prediction. However, adding patient data did not improve performance (AUC = 0.72). A key limitation was the dataset’s lack of variability.

Dembrower et al. (46) focused on using prior mammograms to predict breast cancer risk, comparing a deep learning–based risk score with traditional breast density measures. The mammograms belonged to women served by the Karolinska University hospital and were acquired using Hologic systems. The DL score alone achieved an AUC of 0.65, outperforming dense area (0.60) and percentage density (0.57). The weak correlation between the DL score and density suggests that the model captures non-density-related patterns. However, the authors noted that the model may struggle to generalize to other populations.

## 4 Discussion

The studies reviewed in this paper report promising machine learning performance metrics in the task of BC risk prediction. These findings should be interpreted and discussed cautiously before their clinical impact can be established.

Some of the works reviewed here are inline with the guidelines of European Collaborative on Personalized Early Detection and Prevention of Breast Cancer (ENVISION), which indicates that promising potential in risk prediction might arise from imaging biomarkers. For example, the works of Zhu et al. (22) and Romanov et al. (36), showed to outperform models based on clinical risk factors and traditional risk prediction models, respectively. Despite the positive impacts of image-based AI models, their incorporation in risk prediction should not come at the expense of incorporating established breast cancer risk factors. As discussed in Section 1, BC emergence is related to the presence of several risk factors - some of them inherent to the woman, other related to lifestyle choices. While image-based AI may capture imaging features correlated with some risk factors (such as breast density) it cannot fully account for most of them and their inclusion in combined models might improve accuracy. This is particularly important given the findings of Donnelly et al. (26), where the developed model presents different performance across different race subgroups. Accounting for epidemiological risk factors might help countering this limitation.

The results align with expectations regarding different time frames for risk prediction. The further a mammogram is from a cancer diagnosis, the harder it becomes to predict its appearance accurately. Studies assessing 3-year risk report AUC values between 0.66 and 0.74, whereas those evaluating risk over 5+ years show AUC values at or below 0.66. An exception is the study by Kim et al. (AUC = 0.75); however, as previously discussed, this study has very low sensitivity, limiting its ability to identify high-risk cases effectively. The poor performance of BC risk prediction models over 5+ years may stem from several factors. Although aging generally reduces breast density, some age-related changes may mimic early signs of disease, posing a challenge for risk prediction models. Once again, integrating image-based approaches with common risk factors, such as age and family history, could be beneficial.

Despite variation in performance across studies with different “years-to-cancer” periods, some common conclusions have emerged. For instance, Melek et al. (42), Dadsetan et al. (35), and Wagner et al. (30) all found that incorporating longitudinal or prior mammograms can enhance learning and improve risk prediction. By integrating sequential imaging episodes, models are not limited to analyzing only the current state of a mammogram but can also identify evolving patterns over time. This temporal context provides a more comprehensive learning, leading to more informed decisions and potentially greater predictive accuracy. By analyzing longitudinal imaging data, models may be able to detect more subtle texture changes that are indicative of malignancy, promoting early diagnosis.

Another common finding was the hypothesis that models developed for BC diagnosis might be valuable for risk prediction. This idea was supported by the work of both Santeramo et al. (37) and Lauritzen et al. (45). Their findings suggest an alternative approach to traditional risk prediction research: rather than focusing solely on developing dedicated risk prediction models, researchers could prioritize improving diagnostic models and then adapt them for risk assessment. The advantage of this approach is that diagnostic models are typically trained on well-labeled and easily accessible data. By leveraging this data to identify malignant patterns in mammograms - beyond just lesion detection researchers could enhance risk prediction accuracy while benefiting from the robust foundation of existing diagnostic models. Furthermore, it is worth noting that the limitations reported across studies are highly inconsistent in both scope and level of detail. Some authors describe demographic or technical constraints (e.g., dataset size, ethnicity, equipment vendor), while others report only minimal or no limitations. This lack of uniform reporting complicates the comparison between methods.

When analyzing the types of architectures used, it becomes evident that most studies rely on well-established DL networks, such as ResNet and Inception. Due to their proven effectiveness in various applications. Additionally, several works build upon an existing risk prediction model: MIRAI, with only Melek et al. (42) opting to design a costume model rather than using a pre-trained architecture or an established risk prediction framework.

Despite the promising results obtained by the works reviewed throughout this paper, several limitation must be addressed before these models can be effectively translated into clinical practice. Across all time frames for BC risk prediction, some studies relied on either small datasets, data from a single institution, or images acquired using only one mammography manufacturer, all of which may limit generalizability. Both of these factors hinder a model’s ability to generalize to unseen data, ultimately limiting its clinical utility. Training models on small datasets restricts their exposure to the natural variability of breast tissue across different women. As a result, the models may learn patterns that are not representative of the broader population, leading to decreased performance when applied to new patients. However, simply increasing the number of cases used in model development is not a sufficient solution. If all the data come from the same institution or region, the model remains biased toward the specific characteristics of that population. This includes factors such as regional screening protocols, imaging techniques, and the ethnic composition of the studied group. Therefore, in order to overcome these limitations, it is essential to incorporate diverse, multi-institutional datasets that capture a wide range of imaging variations, screening guidelines, and patient demographics. This would enhance the model’s ability to generalize, ensuring that it performs well across different healthcare settings and populations. Additionally, future studies should prioritize external validation on independent datasets from various regions to further assess the model’s reliability and real-world applicability. Another important limitation in some of the reviewed works relates to the considerable data imbalance in both the training and testing sets. For example, the work of Dembrower et al. (46) includes nearly 2,000 risk-negative cases and only 278 risk-positive cases. A similar pattern is found in the work of Michel et al. (43), with the imbalance skewed toward the negative class. Such imbalance can bias some of the performance metrics used to evaluate the models (particularly accuracy, which is highly sensitive to class imbalance). Nonetheless, imbalanced datasets are often used to mimic real-world conditions, where the prevalence of risk-positive cases is lower than that of risk-negative cases. Given that, the trade-off between realism and methodological rigor must be carefully managed to ensure that the models are both clinically relevant and computationally robust. Finally, in addition to the limitations discussed in this section, one major challenge that could substantially delay the clinical adoption of these models is their nature as “black boxes” (as described by Santeramo et al. (37), for example). For these models to gain acceptance among both clinicians and patients, it is crucial that their predictions are interpretable and transparent (as demonstrated by Donnelly et al. (26), for instance). Providing explanations for the model’s decisions, such as highlighting the specific regions of a mammogram that contribute most to a given prediction, can help clinicians better understand the changes in breast tissue that the model is detecting. This can enhance trust in the system and facilitate its integration into clinical workflows. Additionally, improving explainability can increase patient (and clinician) confidence in AI-driven diagnosis, making them more likely to trust and act upon the results. For these reasons, future efforts should prioritize the development of explainable AI methods Van Zyl et al. (48) like SHAP, which quantifies the contribution of each feature for model output, or GRAD-CAM, originally designed to better understand predictions on image classification by overlaying an heatmap over the input, highlighting important regions. In addition to commonly used explainability methods, future work should focus on the development and deployment of concept-based explainability techniques. These methods aim to better explain the decisions made by the model by using human-understandable concepts or attributes. Such approaches mimic the way humans reason, explain, and make decisions, which may play an important role in increasing clinical acceptance of AI models. However, the development of concept-based methods requires the use of carefully curated datasets, with the definition of concepts tailored to the proposed task - this may be the reason why these methods are not widely adopted Poeta et al. (49). By incorporating interpretability into model designs, researchers can bridge the gap between AI risk prediction models and clinical utility.

By addressing these limitations, these models can further advance the paradigm of personalized medicine, enabling physicians to take proactive measures for women at risk. These measures could range from adapting screening protocols - such as modifying the frequency of screenings of selecting different imaging modalities for early diagnosis - to recommending lifestyle changes to high-risk individuals. Utilizing datasets that represent the diversity observed in screened population, improving generalizability, and improving interpretability can result in AI models becoming a valuable tool in BC risk prediction. By promoting personalized care, these approaches can improve early detection, optimize resource allocation, and ultimately mitigate the effects of this disease.

## 5 Conclusion

The studies reviewed in this paper highlight the immense potential of AI-powered image-based models for BC risk prediction. However, our review is not without limitations. While we aimed to provide an overview of Deep Learning methods for BC risk prediction between 2020 and 2024,we did not conduct a formal risk of bias assessment. This choice was made because we wanted to focus on capturing the full scope of recent developments and identify current trends. Furthermore, even though dividing the studies by risk timeframes helped structure the review, the chosen categorization may not fully represent the diversity of each research.

Nonetheless, it was found that short-term risk models have demonstrated high performance - often surpassing commonly used clinical risk prediction models. However, one major limitation is their ability to generalize across diverse populations. Efforts should be made to improve their adaptability so they can be more effectively integrated into clinical practice.

Additionally, combining imaging biomarkers with traditional clinical risk factors could enhance prediction accuracy while still considering well-established environmental and hereditary factors that influence BC development. Finally, for these AI-based models to gain widespread acceptance in the medical community, they must prioritize explainability - ensuring that their decision-making processes are transparent and interpretable for healthcare professionals and patients.

In addition to ensuring model robustness on unseen data and improve model explainability, clinical acceptance of these AI models also demands a wider view of model reliability. Models should be able to be incorporating irrespective of acquisition protocols, i.e., clinicians should not be expected to completely change their routine when incorporating AI. Moreover, the developed models should also be a able to deal with varying image quality since they can be deployed across different healthcare environments, with different levels of equipment and infrastructure. Addressing all this aspects is essential to ensure a proper acceptance and incorporation of these models into the clinical practice.
